# Identification of PANoptosis-related biomarkers and immune infiltration characteristics in psoriasis

**DOI:** 10.1097/MD.0000000000035627

**Published:** 2023-10-20

**Authors:** Lingling Lu, Buxin Zhang, Meiling Shi, Aimin Liu

**Affiliations:** a Henan University of Chinese Medicine, Zhengzhou, P.R. China; b Department of Dermatology, Henan Province Hospital of Traditional Chinese Medicine, The Second Affiliated Hospital of Henan University of Chinese Medicine, Zhengzhou, P.R. China; c Jinjihu Community Health Service Center of Suzhou Industrial, Suzhou, P.R. China.

**Keywords:** bioinformatics, hub genes, immune microenvironment, PANoptosis, psoriasis

## Abstract

**Background:**

PANoptosis may play a vital role in psoriasis. We investigated the relationship between PANoptosis in psoriasis.

**Methods:**

Genes information was mainly obtained from GeneCards and the gene expression omnibus database. Genefunctions identification was based on gene ontology and Kyoto Encyclopedia of Genes and Genomes analyses. Gene set enrichment analysis was used to identify enriched signaling pathways in psoriasis. We constructed PPI networks using the search tool for the retrieval of interacting genes database and Cytoscape and explored mRNA-miRNA, mRNA-TF, and mRNA-drug interaction networks. Receiver operating characteristic curves were performed to screen potential biomarkers among these hub genes. Immune cell infiltration was analyzed using the Pearson algorithm, and the correlation between immune-cell abundance and PANoptosis-related differentially expressed gene (PDGs) was investigated.

**Results:**

We identified 10 PDGs, which were mainly involved in pyroptosis, cytokine-mediated signaling pathways, Salmonella infection and NOD-like receptor signaling pathway. The activated pathways were mostly proinflammatory and immunoregulatory pathways between immune cells. BAK1, CASP4, IL18, and IRF1 were identified as hub genes in the mRNA-miRNA network, and BAK1, IRF1, and PYCARD were hub genes in the mRNA-TF network. CASP1 was found to be the most targeted gene by drugs or molecular compounds. We found PDGs were positively associated with proinflammatory immune cell infiltration and negatively associated with anti-inflammatory or regulatory immune cells.

**Conclusion:**

We confirmed the role of PANoptosis in psoriasis for the first time and predicted hub genes and immune characteristics, which provides new ideas for further investigation of psoriasis on pathogenic mechanisms and therapeutic strategies.

## 1. Introduction

Psoriasis is a common chronic inflammatory skin disease, which affects approximately 1% to 3% population worldwide.^[1]^ Typical characteristics of the disease are erythematous papules and plaques with a silver scale.^[2]^ The pathogenesis of psoriasis is complex and the exact etiology of the disease is still unknown. Interleukin- (IL-) 23/IL-17 axis is crucial to the development of psoriasis.^[3]^ The inflammation of psoriatic skin is considered a result of abnormal communication between infiltrating immune cells and activated keratinocytes (KC). Notably, psoriasis is highly associated with several important medical conditions, such as hypertension, and cardiovascular diseases.^[4]^ Patients with the disease have been shown to be at increased risk for mental health issues. They might feel embarrassed about the appearance of their skin, and experience feelings of stigmatization.^[5]^ They are also at a higher risk of developing depression, anxiety, social phobia, and addiction (e.g., smoking and alcohol), even suicidal ideation.^[6,7]^ Biologics have been demonstrated to be highly beneficial for the initial treatment of psoriasis, however, responsiveness might decline over time, resulting in treatment discontinuation or changing. Understanding the etiology and pathophysiology of psoriasis could thereby guide therapeutic therapy, improving clinical results and patient life quality.

Programmed cell death (PCD) is a key component of the innate immune response. To date, the most well-defined and studied PCD pathways include apoptosis, necroptosis, and pyroptosis.^[8]^ Recently, a highly interconnected PCD, termed PANoptosis, has been defined. PANoptosis is an inflammatory PCD pathway regulated by the PANoptosome complex, which includes the mixture features of pyroptosis, apoptosis, and/or necroptosis and essential molecules of the 3 pathways. The relation of PANoptosis with psoriasis remains largely unclear, however, accumulated evidence demonstrates that PANoptosis is implicated in psoriasis pathogenesis. Programmed apoptosis,^[9]^ necrosis^[10]^ and pyroptosis^[11]^ were confirmed to be correlated with psoriasis inflammation. Meanwhile, several psoriasis-associated proteins have been identified in PANoptosis, and have essential roles in cell survival and inflammatory immune signaling pathways. For example, AIM2, an important member of the PANoptosome complex, has been shown to be elevated in KC of psoriatic lesions and could increase the release of IL-1β and IL-18 upon stimulation, which consequently activates the IL-23/IL-7 axis and exerts a pro-inflammatory effect.^[12]^

The IL-23/IL-17 axis plays an essential role in the development of psoriasis and is also strongly associated with PANoptosis. In psoriasis, triggering factors like trauma and infection could stimulate IL-23 production and consequently induce Th17 cells to produce pro-inflammatory cell cytokines like IL-17, which could in turn amplify the inflammatory cascade response and exacerbate skin inflammation.^[13]^ Furthermore, there are also interactions between IL-23/Il-17 axis and PCD signaling pathways, which could ultimately induce PANoptosis and strong inflammatory response. Specifically, the role of IL-17-mediated activation of the NLRP3 inflammasome complex in psoriasis inflammation is widely demonstrated,^[14,15]^ meanwhile, the activation of NLRP3 inflammasome complex is also closely related to cell death like pyroptosis, apoptosis and necrosis, which ultimately leads to elevated production of IL-1β and IL-17.^[16]^ Triggers like infection can also activate IL-23/IL-17-mediated immune responses by inducing caspase-mediated apoptosis of KC.^[17]^ Necrosis could promote IL-17 expression and contribute to psoriasis as well. In psoriasis lesions, the expression of necroptosis-related molecules like RIPK1 and RIPK3 was significantly increased, and the activation of necroptosis mediated by RIPK1/RIPK3 pathway showed a significant effect on the upregulation of IL-17 and could subsequently induce chronic inflammation in psoriasis.^[10]^ IL-23/IL-17 axis is also closely related to the PANoptosome complex. A20, a component of PANoptosome, exerts an anti-inflammatory effect by interfering the activation of PANoptosome.^[8]^ Researchers found A20 may reduce inflammation in psoriasis by restricting IL-23/IL-17 pathway.^[18]^ Taken together, PANoptosis might be closely coupled with the pathogenesis of psoriasis. These above-mentioned interactions between IL-23/IL-17 signal and PANoptosis could ultimately lead to amplified inflammatory cascade response, which plays a crucial role in the development of psoriasis. Nonetheless, the exact mechanisms of PANoptosis underlying psoriasis remain indistinct. Consequently, we performed the study with the aim of clarifying whether PANoptosis-related genes (PRGs) could be accurate biomarkers for psoriasis and their effects on the immune microenvironment by bioinformatics analysis.

With the aim of clarifying the involvement of PANoptosis in psoriasis and the underlying molecular mechanisms. We analyzed gene expression data sets from the gene expression omnibus (GEO) database, data in genecards database and the PubMed website. The determination of PANoptosis-related differentially expressed genes (PDGs) in psoriasis and the underlying mechanisms was conducted with a series of bioinformatics analysis and enrichment analysis. Protein-protein interaction (PPI) network establishment and identification of hub genes using search tool for the retrieval of interacting genes (STRING) database and Cytoscape software. The diagnostic accuracy of potential targeted genes was examined with receiver operating characteristic (ROC) curve analyses. Additionally, the relationship between PDGs in psoriasis and immune-cell infiltration was also investigated.

## 2. Materials and methods

### 2.1. Data source

In this study, the expression profile data sets of psoriasis patients including GSE6710,^[3]^ GSE14905,^[4]^ and GSE30999^[5,6]^ were downloaded from GEO (https://www.ncbi.nlm.nih.gov/geo) database with “GEO query” package in R. All data in these datasets were form Homo sapiens. GSE6710 was based on GPL96 HG-U133A Affymetrix Human Genome U133A Array, GSE14905 and GSE30999 were based on GPL570 HG-U133_Plus_2 Affymetrix Human Genome U133 Plus 2.0 Array. Samples in both GSE6710 and GSE30999 were all microarray gene-expression profiles of psoriasis lesion skin (LS) and non-lesion (NL) from 13 psoriasis patients. Samples in GSE14905 were microarray gene-expression profiles of LS and NL from psoriasis patients, as well as normal skin tissues from healthy individuals. For the subsequent analysis, all the expression profiles of psoriasis patients in GSE6710 and 33 LS and 28 NL samples in GSE14905 were chosen. Concerning GSE14905, all the expression profile data of 85 LS and 85 NL samples from psoriasis patients were included for analysis. All data used in the study were public data, and hence, ethics approval was not required.

PANoptosis is an important type of cell death that has received increasing attention recently. The GeneCards^[19]^ database provides comprehensive information on human genes. We obtained the PRGs from the GeneCards database, with the term of “PANoptosis,” and kept only “Protein Coding” and “Relevance score > 0.500” genes. Additionally, we searched for literatures^[20–22]^ on the PubMed website to identify relevant PRGs. In total, 12 PRGs in GeneCards and 60 in published literature from the PubMed website were identified. After merging, and deduplication, 62 PRGs were retained.

### 2.2. Identification of PDGs

To investigate the probable mechanism, biological features, and pathways of differentially expressed genes (DEGs) in psoriasis, the limma^[23]^ package was used to normalization and annotation the 3 datasets. After data preprocessing, a DEGs list between psoriasis and normal samples was obtained. Only those with adjusted P.adj value < 0.05 and |logFC| > 0 were regarded as DEGs. If logFC > 0 and P.adj < 0.05, DEGs were considered upregulated, those with logFC < 0 and P.adj < 0.05 were considered downregulated. After that, we constructed a Venn diagram of all DEGs of 3 datasets to find the common DEGs (Co-DEGs). Finally, the PDGs were generated by intersecting Co-DEGs and PRGs using a Venn diagram. Volcano map and heat map were conducted based on “ggplot2” and “pheatmap” package in R, respectively.

### 2.3. Gene and functional enrichment analysis of DEGs.

Gene ontology (GO)^[24]^ analysis is widely used in functional enrichment researches of large number of gene, since it gives straightforward annotations of gene products on aspects of functions, biological pathways involved, and cell location. Kyoto encyclopedia of genes and genomes (KEGG)^[25]^ is a widely used database that containing information on genomes, biological pathways, diseases, and chemical substances. ClusterProfiler R package^[26]^ were used to conduct gene annotation enrichment analysis about PRGs. *P* value < .05 and false discovery rate (FDR) (*q*.value) < 0.05 were regarded as statistically significant. The Benjamini–Hochberg adjustment was applied to correct *P* values.

### 2.4. Gene set enrichment analysis

Gene set enrichment analysis (GSEA)^[27]^ assesses the distribution of predefined gene sets throughout gene lists sorted by phenotypic relevance to measure the contribution of genes to phenotype. First, we divided the genes in GSE6710, GSE14905 and GSE30999 datasets into psoriasis group and normal group according to the severity of the disease. Next, we used the clusterProfiler to conduct functional enrichment evaluations on all DEGs in the two groups in accordance with phenotypic relevance. The following are the parameters utilized in this GSEA: the seed was 2022, the number of computations is 10,000. Each gene set had at least 10 and no more than 500 genes. *P* value was corrected by the Benjamini–Hochberg method. GSEA was conducted using MSigDB collections of “c2.cp.all.v202212.Hs.symbols.gmt.” P.adj < 0.05 and FDR (*q*.value) < 0.05 indicated significantly enriched.

### 2.5. PPI network analysis

The PPI network is made up of interacting proteins and involved in biological signaling, gene expression regulation, energy and substance regulation and other life processes. A systematic analysis of protein interactions in biological systems is highly beneficial for understanding the protein biological mechanisms, the response mechanisms of biochemical signals, materials and energy in certain physiological situations, as well as the connections and functional interactions between proteins. The STRING^[28]^ database is utilized to identified interactions between known and hypothetical proteins. We created PPI network for PDGs using the database, and set the minimum interaction score to medium confidence (0.400), and visualized the PPI network using Cytoscape^[29]^ (version 3.9.1) and recognized these PDGs as psoriasis hub genes.

### 2.6. Construction of mRNA-miRNA, mRNA-TF,and mRNA-drugs interaction networks

Potential miRNA targets could be obtained using the online database miRDB (http://www.mirdb.org), along with functional annotations.^[30]^ In the database, we obtained miRNAs that could potentially interact with hub genes, selected those with target score >80 and established mRNA-miRNA regulatory network. The CHIPBase^[31]^ (https://rnasysu.com/chipbase3/index.php) is an online resource for studying ChIP-seq data. It could be used to identify potential regulatory elements and target genes of DNA-binding proteins and predict the interaction relationship between transcription factors (TF) and genes. The hTFtarget database^[32]^ (http://bioinfo.life.hust.edu.cn/hTFtarget) contains human TF and corresponding regulatory targets. We obtained TF that combined with hub genes based on the above databases, and used Cytoscape to visualize networks. The Drug Gene Interaction Database^[33]^ were examined to obtain potential medications or small molecule substances interacting with hub genes. The visualization of regulatory networks was constructed by Cytoscape.

### 2.7. ROC curve

The ROC curve is a fundamental analytical tool that may be applied to choose most appropriate model or to determine the optimal threshold within a model.^[34]^ The ROC curve is an integrated index reflecting the continuous variables of sensitivity and specificity, and presents the interrelation of sensitivity and specificity using composition method. The area under the ROC curve (AUC) is typically ranges from 0.5 to 1. When the AUC approaches 1, the diagnostic performance is considered better. In cases where the AUC falls between 0.5 and 0.7, the accuracy is low; for values between 0.7 and 0.9, the accuracy is moderate; and for values above 0.9, the accuracy is high. We utilized the pROC package to produce ROC curves for PDGs and calculate the AUC to assess the PDGs diagnostic efficacy on survival.

### 2.8. Immune infiltration analysis (CIBERSORT)

The CIBERSORT^[35]^ algorithm is an analytical tool for estimating immune cell infiltration. It uses linear support vector regression to separate the transcriptome matrix and determine the components and quantity of immune cells in mixed cell populations. We screened out the data with an enrichment value of immune cells greater than zero using the LM22 characteristic gene matrix along with the PDGs matrix data in the psoriasis data set through the CIBERSORT package,^[36]^ and then we obtained and exhibited the precise findings about the abundance matrix of immune cell infiltration.

The content of immune cells in samples was illustrated in boxplot. The interrelationship between immune cells and PDGs was created by spearman algorithm and the correlation heatmap was conducted by pheatmap package.

### 2.9. Statistical analysis

R software (Version 4.1.2) was employed to process and analysis data. Continuous variables are presented as means ± SD. The Wilcoxon rank sum test was utilized to compare difference between 2 groups. Comparisons of more than 3 groups were performed with the Kruskal–Wallis test. If not specified specifically, Spearman correlation analysis was employed to figure out correlation coefficients between different compounds. A significant difference was considered when the *P* value < .05.

## 3. Results

### 3.1. Standardization of data sets

**Figure 1.**
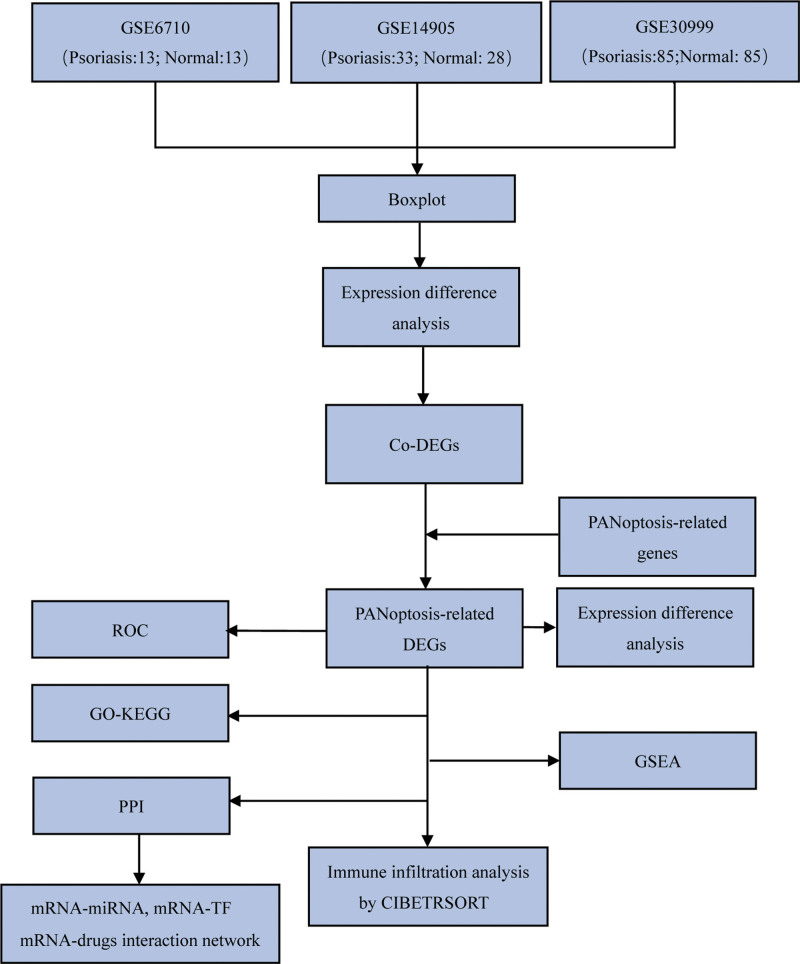
Flowchart. DEG = differentially expressed genes; Co-DEGs = common differentially expressed genes; GO = gene ontology; KEGG = Kyoto encyclopedia of genes and genomes; GSEA = gene set enrichment analysis; PPI = protein-protein interaction; TF = transcription factors; ROC = receiver operating characteristic curve.

**Figure 2.**
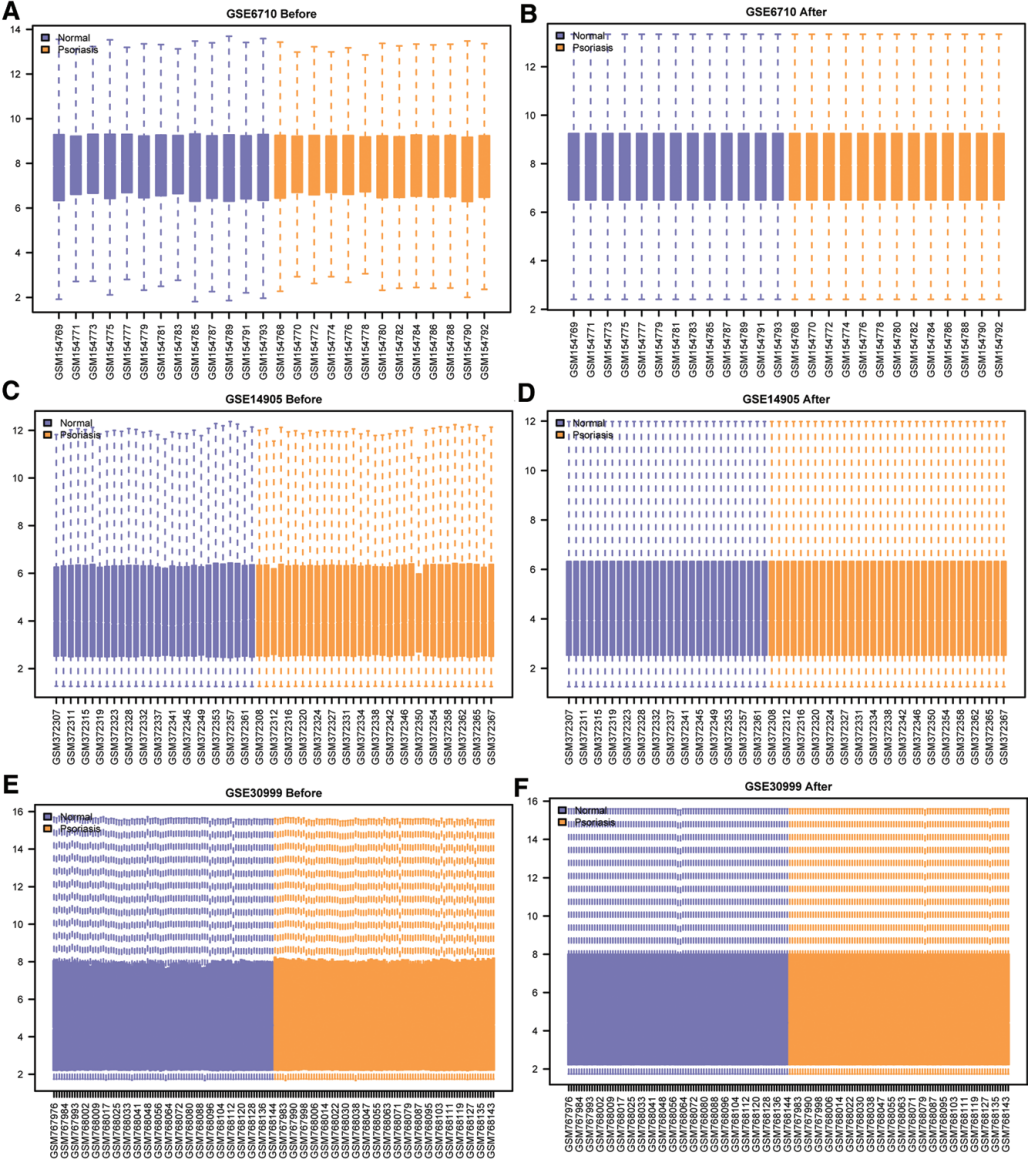
Normalization of the Psoriasis dataset. Boxplot plots of the GSE6710 dataset before (A) and after (B) normalization. (C and D) boxplot of the GSE14905 dataset before (C) and after (D) normalization. (E and F) boxplot of the GSE30999 dataset before (E) and after (F) normalization.

In total, we gathered 3 data sets: GSE6710, GSE14905and GSE30999 (Table 1). In GSE6710, there were 26 samples with 13 LS and 13 NL samples from 13 psoriasis patients. GSE14905 contains 82 samples, including 33 LS and 28 NL from psoriasis patients, along with 21 normal skin samples from healthy individuals. GSE30999 dataset includes 170 samples with 85 LS samples and 85 NL. Totally, we retrieved 12 PRGs from the GeneCards database and 60 PRGs from literatures. After merging the results and removing duplicates, 62 PRGs were obtained. The flowchart of is shown in Figure 1. We normalized the 3 psoriasis datasets using the R package limma package, respectively (Fig. 2A–F). In GSE6710 dataset, there were 26 samples, including 13 normal samples and 13 psoriasis disease samples (Fig. 2A and B). In the GSE14905 dataset, there were 61 samples, including 28 normal samples and 33 psoriasis samples (Fig. 2C and D). The GSE30999 dataset covers 170 samples, 85 samples in each group (Fig. 2E and F). The results demonstrated that the batch effect between samples of each dataset was basically removed after standardized processing, and data distributions between each dataset tended to be consistent. (Fig. 2A–F)

**Table 1:** List of Psoriasis datasets information.

	GSE6710	GSE14905	GSE30999
Platform	GPL96	GPL570	GPL570
Species	Homo sapiens	Homo sapiens	Homo sapiens
Tissue	Skin biopsy	Skin biopsy	Skin biopsy
Samples in Psoriasis group	13	33	85
Samples in Normal group	13	28	85
Reference	Increased expression of Wnt5a in psoriatic plaques.	Type I interferon: potential therapeutic target for psoriasis?	Expanding the psoriasis disease profile: interrogation of the skin and serum of patients with moderate-to-severe psoriasis. Shrinking the Psoriasis Assessment Gap: Early Gene-Expression Profiling Accurately Predicts Response to Long-Term Treatment.

BP = biological process, CC = cellular component, DEG = differentially expressed genes, GO = gene ontologys, MF = molecular function.

### 3.2. Identification of PDGs

**Figure 3.**
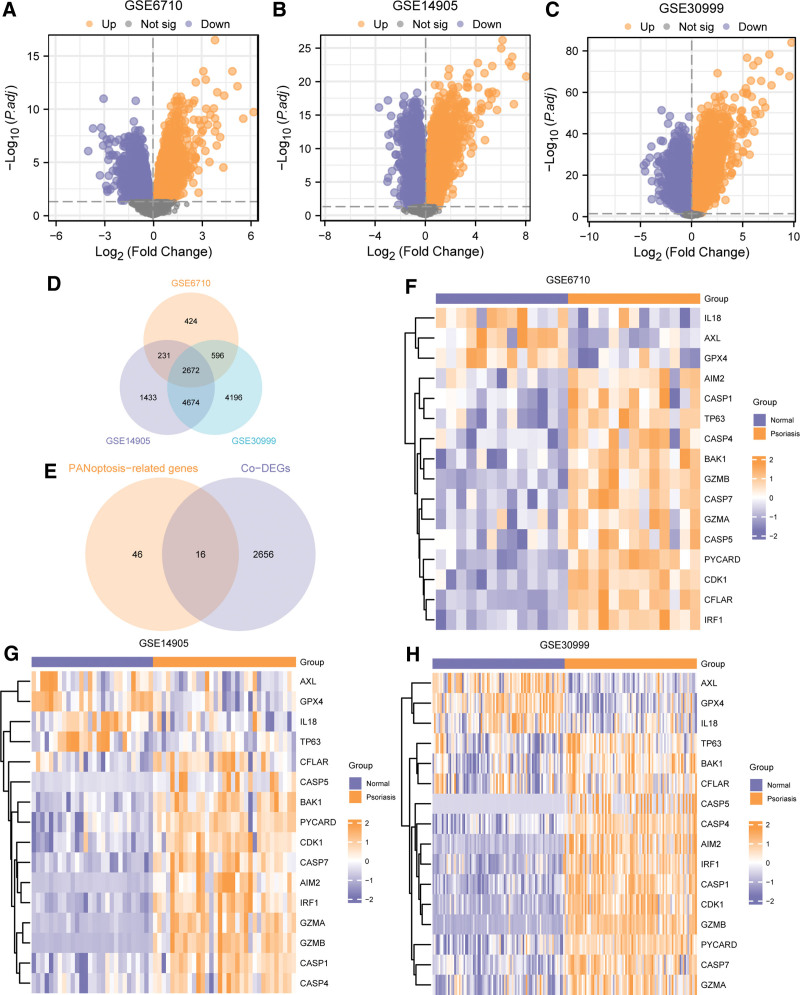
DEGs analysis of psoriasis and heatmap of PDGs. Volcano plot showing DEGs in GSE6710(A), GSE14905(B) and GSE3099(C), Venn diagram shows the Co-DEGs of 3 datasets (D) and PDGs in psoriasis (E), the heatmap displayed the expression of PDGs in psoriasis between psoriasis and normal group in GSE6710(F), GSE14905(G) and GSE3099(H). DEGs = differentially expressed genes; Co-DEG = common differentially expressed genes; PDGs = PANoptosis-related differentially expressed genes.

We used the limma package to normalize the psoriasis and normal data in the GSE6710, GSE14905 and GSE30999, and obtained the DEGs between the psoriasis and the normal group. The results were as follows: 3937 DEGs were obtained in the GSE6710 dataset based on criterion that P.adj < 0.05 and logFC > 0. Totally, we got 1914 upregulated and 2023 downregulated genes in psoriasis group, and presented the results were in the volcano plot (Fig. 3A). In GSE14905, there were 21655 DEGs. Finally, 9019 were considered eligible, which included 4007 upregulated DEGs and 5012 downregulated DEGs. The results were presented in Figure 3B. In GSE30999, 21655 DEGs were obtained, and a total of 12156 DEGs met the mentioned criteria, 5790 genes were upregulated and 6366 were downregulated. The results were illustrated in Figure 3C. In order to obtain PDGs, we firstly took the intersection of all DEGs meeting the criteria that |logFC| > 0 and P.adj < 0.05 in the 3 databases, then acquired 2672 Co-DEGs and constructed the Venn diagram (Fig. 3D). Then we took the intersection between Co-DEGs and PRGs, and obtained 16 PDGs in psoriasis (Fig. 2E), namely, AIM2, AXL, BAK1, CASP1, CASP4, CASP5, CASP7, CDK1, CFLAR, GPX4, GZMA, GZMB, IL18, IRF1, PYCARD, and TP63. According to the results of the Venn diagram, we explored the expression difference of the 16 PDGs between psoriasis and the normal group in the GSE6710 (Fig. 3F), GSE14905 (Fig. 3G), and GSE30999 (Fig. 3H), respectively, and presented the results in heatmaps based on the pheatmap package.

### 3.3. Functional annotation of PDGs in psoriasis

**Figure 4.**
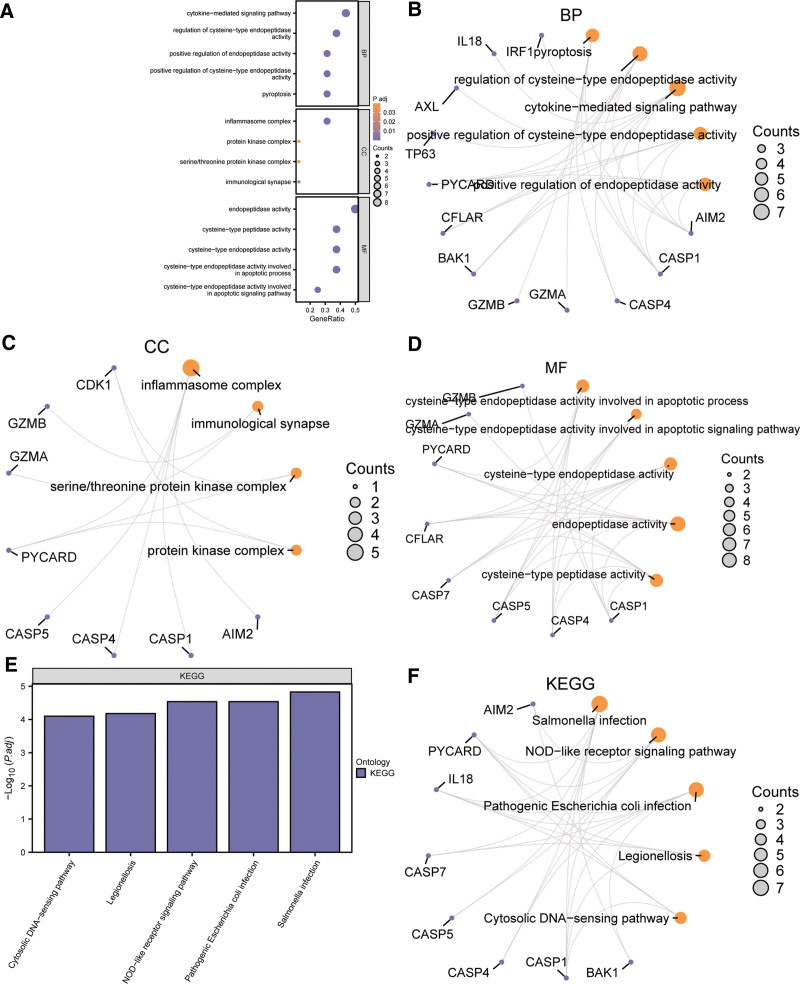
GO and KEGG analysis of PANoptosis-related differentially expressed gene (PDGs). (A) The results of GO enrichment analysis were presented in bubble plots, (B and C) Network diagram of BP (b), CC (c), and MF (d). (E and F) Results of KEGG analysis presented in bar graph (E) and network diagram (F). In the bubble plot (A), the ordinate is the GO terms, and the bubble color indicates the Padj - value. In the network diagram (B, C, D, F), purple dots represent specific genes and orange circles represent specific pathways. The screening criteria for GO and KEGG enrichment were *P* value < .05 and false discovery rate (FDR) value (*q*. value) < 0.05. BP = biological process; CC = cellular component; GO = gene ontology; KEGG = Kyoto encyclopedia of genes and genomes; MF = molecular function.

To explore the biological process (BP), molecular function (MF), cellular component (CC) and biological pathways of the 16 PDGs in psoriasis, GO functional analysis (Table 2) together with KEGG pathway (Table 3) analysis were carried out. According to the results, BP of the 16 PDGs were mainly enriched in 5 GO terms, including pyroptosis, regulation of cysteine-type endopeptidase activity, cytokine-mediated signaling pathway, positive regulation of cysteine-type endopeptidase activity, positive regulation of endopeptidase activity, respectively. CC mainly accounted for 4 GO terms including inflammasome complex, protein kinase complex, serine/threonine protein kinase complex and immunological synapse, and MF accounted for 5 GO terms like endopeptidase activity, cysteine-type peptidase activity, cysteine-type endopeptidase activity, cysteine-type endopeptidase activity involved in the apoptotic process, cysteine-type endopeptidase activity involved in apoptotic signaling pathway. The results of GO functional were demonstrated by bubble diagram (Fig. 4A). In addition, we showed the results of BP, CC, and MF in the form of a network diagram (Fig. 4B–D).

**Table 2:** GO enrichment analysis results of PANoptosis-related DEGs.

Ontology	ID	Description	GeneRatio	BgRatio	*P* value	p.adjust
BP	GO:0070269	pyroptosis	5/16	22/18800	5.83e-12	5.72e-09
BP	GO:2000116	regulation of cysteine-type endopeptidase activity	6/16	229/18800	2.21e-08	1.08e-05
BP	GO:0019221	cytokine-mediated signaling pathway	7/16	486/18800	6.91e-08	2.26e-05
BP	GO:2001056	positive regulation of cysteine-type endopeptidase activity	5/16	143/18800	9.69e-08	2.37e-05
BP	GO:0010950	positive regulation of endopeptidase activity	5/16	174/18800	2.58e-07	5.05e-05
CC	GO:0061702	inflammasome complex	5/16	18/19594	1.55e-12	6.34e-11
CC	GO:0001772	immunological synapse	2/16	44/19594	.0006	0.0119
CC	GO:1902554	serine/threonine protein kinase complex	2/16	99/19594	.0029	0.0396
CC	GO:1902911	protein kinase complex	2/16	115/19594	.0039	0.0398
MF	GO:0097153	cysteine-type endopeptidase activity involved in apoptotic process	6/16	15/18410	7.39e-16	4.58e-14
MF	GO:0097199	cysteine-type endopeptidase activity involved in apoptotic signaling pathway	4/16	10/18410	7.96e-11	2.47e-09
MF	GO:0004197	cysteine-type endopeptidase activity	6/16	120/18410	5.13e-10	1.06e-08
MF	GO:0004175	endopeptidase activity	8/16	432/18410	9.41e-10	1.46e-08
MF	GO:0008234	cysteine-type peptidase activity	6/16	178/18410	5.55e-09	6.88e-08

BP = biological process, CC = cellular component, DEG = differentially expressed genes, GO = gene ontology, MF = molecular function.

**Table 3:** KEGG enrichment analysis results of PANoptosis-related DEGs.

Ontology	ID	Description	GeneRatio	BgRatio	*P* value	p.adjust
KEGG	hsa05132	Salmonella infection	7/16	249/8164	2.04e-07	1.49e-05
KEGG	hsa04621	NOD-like receptor signaling pathway	6/16	184/8164	8.02e-07	2.91e-05
KEGG	hsa05130	Pathogenic Escherichia coli infection	6/16	197/8164	1.2e-06	2.91e-05
KEGG	hsa05134	Legionellosis	4/16	57/8164	3.65e-06	6.66e-05
KEGG	hsa04623	Cytosolic DNA-sensing pathway	4/16	63/8164	5.47e-06	7.98e-05

DEG = differentially expressed genes, KEGG = Kyoto encyclopedia of genes and genomes.

Regarding KEGG, the 16 PDGs were mainly enriched in 5 pathways, including Salmonella infection, NOD-like receptor signaling pathway, Pathogenic Escherichia coli infection, Legionellosis, Cytosolic DNA-sensing pathway. The results were presented in a bar graph (Fig. 4E) and a network diagram (Fig. 4F).

### 3.4. GSEA

**Figure 5.**
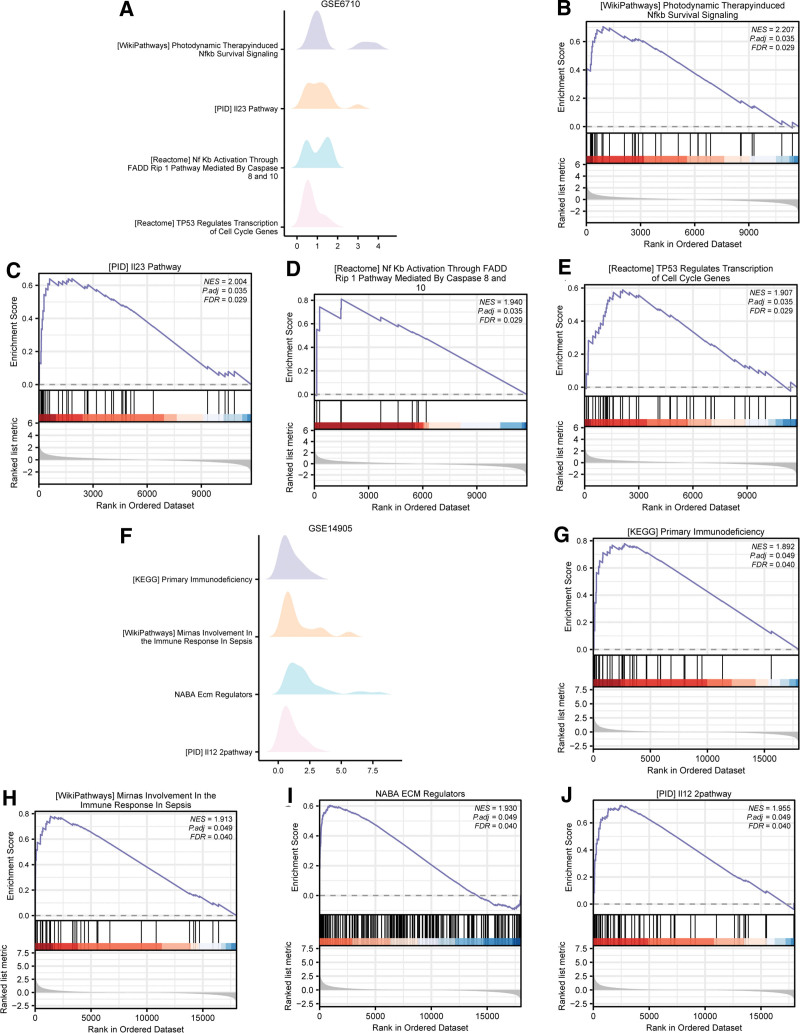
GSEA of GSE6710 and GSE149054. (A) Four main biological features of GSE6710 dataset from GSEA enrichment analysis. (B–E) differentially expressed genes (DEGs) in GSE6710 dataset were enriched in pathways of photodynamic therapy induced NF-KB survival signaling (B), IL23 pathway (C), and NF-KB activation through FADD RIP1 pathway mediated by caspase 8 and 10 (D), TP53 regulates transcription of cell cycle genes (E). (F) Four main biological features of GSE14905 dataset from GSEA enrichment analysis. The DEGs were significantly enriched in primary immunodeficiency (G), Mirnas involvement in the immune response in sepsis (H), NABA ECM regulators (I) and I12 2pathway (J), The screening criterion of GSEA was *P *< .05 and false discovery rate (FDR) value < 0.05. GSEA = gene set enrichment analysis.

To figure out the interrelationship between DEGs and psoriasis, GSEA was conducted to explore the expression of DEGs, as well as BP, CC, and MF of these genes in GSE6710, GSE14905 and GSE30999 datasets, respectively. *P *< .05 and FDR value (*q.*value) < 0.05 was used as the screening criterion for significant enrichment. We found DEGs in GSE6710 were primarily concentrated in 4 biological characteristics (Fig. 5A and Table 4), they were photodynamic therapy induced NF-kb survival signaling (Fig. 5B), IL23 pathway (Fig. 5C), NF-KB activation through FADD RIP1 pathway mediated by caspase 8 and 10 (Fig. 5D), TP53 regulates transcription of cell cycle genes (Fig. 5E). In GSE14905, the DEGs were mainly enriched in 4 pathways (Fig. 5F, Table 5), namely, primary immunodeficiency (Fig. 5G), Mirnas involvement in the immune response in sepsis (Fig. 5H), NABA ECM regulators (Fig. 5I) and I12 2 pathway (Fig. 5J).

**Table 4:** GSEA analysis of dataset GSE6710.

Description	setSize	enrichmentScore	NES	p.adjust	qvalues
WP_PHOTODYNAMIC_THERAPYINDUCED_NFKB_SURVIVAL_SIGNALING	34	0.705359	2.207175	0.002012	0.034575
PID_IL23_PATHWAY	35	0.64011	2.003594	0.002037	0.034575
REACTOME_NF_KB_ACTIVATION_THROUGH_FADD_RIP_1_PATHWAY_MEDIATED_BY_CASPASE_8_AND_10	11	0.809466	1.940491	0.001942	0.034575
REACTOME_TP53_REGULATES_TRANSCRIPTION_OF_CELL_CYCLE_GENES	42	0.589335	1.907008	0.001988	0.034575
REACTOME_DECTIN_1_MEDIATED_NONCANONICAL_NF_KB_SIGNALING	56	0.532501	1.837955	0.001969	0.034575
BIOCARTA_MAPK_PATHWAY	80	0.495748	1.826556	0.001938	0.034575
REACTOME_TNFR2_NON_CANONICAL_NF_KB_PATHWAY	89	0.487125	1.825896	0.001965	0.034575
PID_IL12_2PATHWAY	58	0.501848	1.747307	0.002012	0.034575
WP_IL18_SIGNALING_PATHWAY	244	0.346353	1.486301	0.001972	0.034575
REACTOME_TRANSCRIPTIONAL_REGULATION_BY_TP53	295	0.326591	1.434934	0.001957	0.034575

GSEA, gene set enrichment analysis.

**Table 5:** GSEA analysis of dataset GSE14905.

Description	setSize	enrichmentScore	NES	p.adjust	qvalues
PID_IL12_2PATHWAY	60	0.729032	1.955309	0.001724	0.048722
NABA_ECM_REGULATORS	226	0.604081	1.92975	0.001468	0.048722
WP_MIRNAS_INVOLVEMENT_IN_THE_IMMUNE_RESPONSE_IN_SEPSIS	36	0.779567	1.913128	0.001832	0.048722
KEGG_PRIMARY_IMMUNODEFICIENCY	35	0.778851	1.892291	0.001842	0.048722
WP_IMMUNE_RESPONSE_TO_TUBERCULOSIS	23	0.829714	1.884619	0.001815	0.048722
REACTOME_DECTIN_1_MEDIATED_NONCANONICAL_NF_KB_SIGNALING	58	0.683126	1.824529	0.00173	0.048722
REACTOME_FCERI_MEDIATED_NF_KB_ACTIVATION	79	0.647208	1.821693	0.001669	0.048722
REACTOME_HEDGEHOG_LIGAND_BIOGENESIS	60	0.674482	1.809002	0.001724	0.048722
REACTOME_TNFR2_NON_CANONICAL_NF_KB_PATHWAY	95	0.615294	1.781397	0.001637	0.048722
REACTOME_IMMUNOREGULATORY_INTERACTIONS_BETWEEN_A_LYMPHOID_AND_A_NON_LYMPHOID_CELL	124	0.542921	1.61733	0.001631	0.048722

GSEA, gene set enrichment analysis.

**Figure 6.**
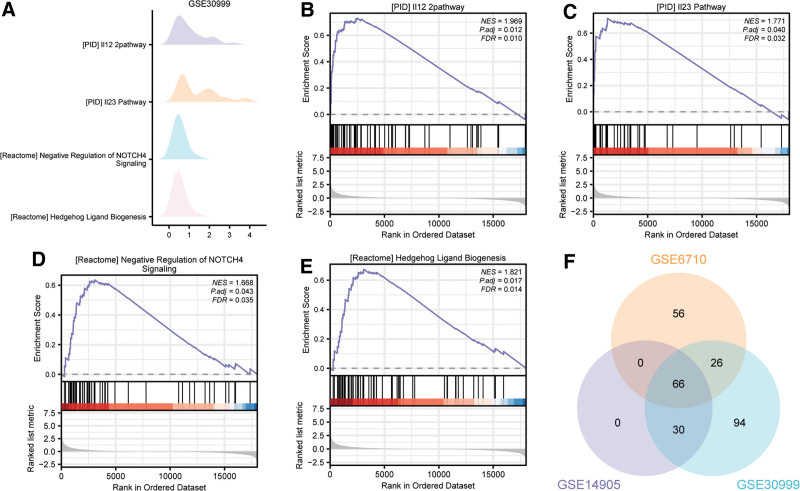
GSEA of GSE30999. (A) Four main biological features of GSE30999 dataset from GSEA enrichment analysis. (B–E) differentially expressed genes (DEGs) in GSE30999 dataset were enriched in pathways of IL12 2pathway (B), IL23 pathway (C), negative regulation of notch4 signaling (D), and hedgehog ligand biogenesis (E). The screening criterion of GSEA was *P *< .05 and false discovery rate (FDR) value < 0.05. (F). Overlaps of significantly enriched pathways from GSEA in 3 datasets. GSEA = gene set enrichment analysis. The screening criterion of GSEA was *P *< .05 and FDR value < 0.05.

In GSE30999, the DEGs were enriched in 4 pathways (Fig. 6A, Table 6), including I12 2pathway (Fig. 6B), IL23 pathway (Fig. 6C), regulation of notch4 signaling (Fig. 6D), hedgehog ligand biogenesis (Fig. 6E). Furthermore, we intersected all functional pathways significantly enriched by GSEA in the 3 datasets and plotted Venn diagram (Fig. 6F). Finally, we obtained 66 functional pathways co-enriched in the 3 datasets, including interferon alpha beta signaling, sarscov2 innate immunity evasion and cell specific immune response, cell cycle checkpoints.

**Table 6:** GSEA analysis of dataset GSE30999.

Description	setSize	enrichmentScore	NES	p.adjust	qvalues
PID_IL12_2PATHWAY	60	0.729032432	1.96872633	0.000348675	0.011936001
PID_IL23_PATHWAY	37	0.713133255	1.77119086	0.002883922	0.039954308
REACTOME_NEGATIVE_REGULATION_OF_NOTCH4_SIGNALING	51	0.635810043	1.667818215	0.00353857	0.04279081
REACTOME_HEDGEHOG_LIGAND_BIOGENESIS	60	0.674482097	1.821415079	0.00069735	0.016783794
REACTOME_DECTIN_1_MEDIATED_NONCANONICAL_NF_KB_SIGNALING	58	0.683126247	1.836467887	0.000347826	0.011936001
REACTOME_FCERI_MEDIATED_NF_KB_ACTIVATION	79	0.647208426	1.821001539	0.000340483	0.011936001
REACTOME_TNFR2_NON_CANONICAL_NF_KB_PATHWAY	95	0.615293508	1.782989498	0.000331455	0.011936001
REACTOME_SWITCHING_OF_ORIGINS_TO_A_POST_REPLICATIVE_STATE	87	0.64417824	1.8484835	0.000332447	0.011936001
REACTOME_CELLULAR_RESPONSE_TO_HYPOXIA	69	0.609172601	1.683032695	0.002401372	0.035861456
REACTOME_APOPTOSIS	163	0.481870034	1.496165533	0.003358779	0.042711555

GSEA = gene set enrichment analysis, NES = normalized enrichment score.

### 3.5. Hub genes and regulatory networks

**Figure 7.**
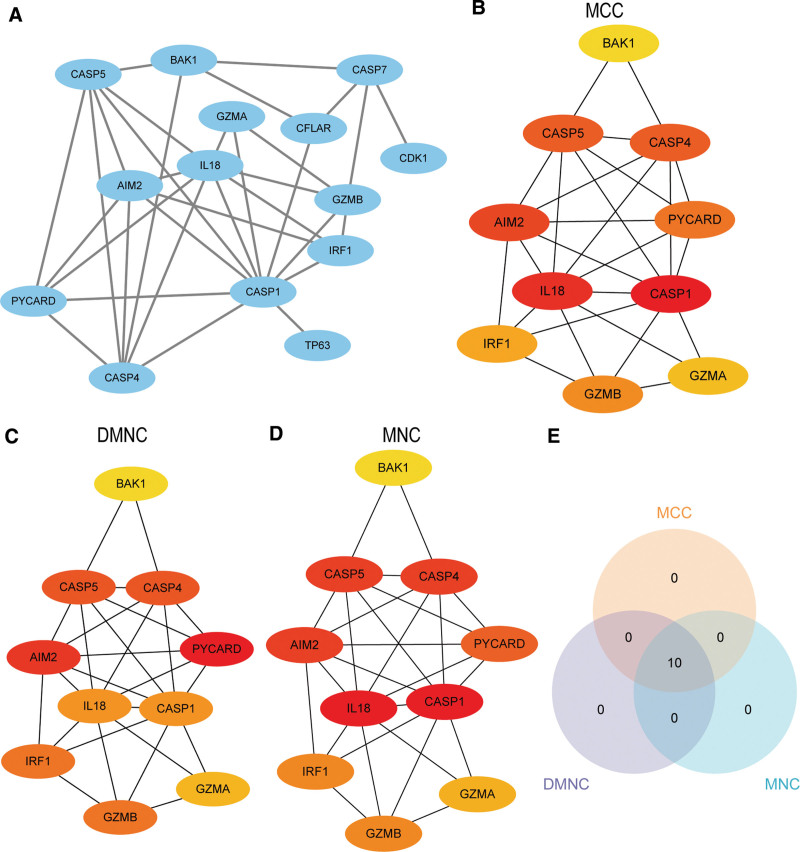
PPI network of PDGs. (A) PPI network of PDGs. B-D. The PPI network of top 10 PDGs obtained by MCC (B), DMNC (C), and MNC (D) algorithms, the color of the dot blocks in the figure from yellow to red represents the gradually increased score. E. Intersection of top 10 PDGs of MCC, DMNC, and MNC algorithms. DMNC: differential metabolic network construction; MCC: Matthews correlation coefficient metric; MNC: the maximal neighborhood coefficient; PDGs = PANoptosis-related differentially expressed genes; PPI network = protein-protein interaction network.

STRING database were used to perform PPI network analysis of the 16 PDGs. All parameters in STRING database were remained the default values, the minimum interaction score was set to medium confidence (0.400). We also established PPI network of 14 PDGs. Cytoscape was used to visualize the network (Fig. 7A). We used the cytoHubba in Cytoscape to calculate the PPI network using MCC, DMNC, and MNC algorithms and selected the top 10 PDGs with the highest scores (Fig. 7B–D). They were AIM2, BAK1, CASP1, CASP4, CASP5, GZMA, GZMB, IL18, IRF1, and PYCARD, respectively. The color of the dot blocks in the figure from yellow to red represents the gradual increase in the score. Then we took the intersection of the top 10 PDGs obtained by the 3 algorithms to obtain the hub genes for psoriasis and presented the results in a Venn diagram (Fig. 7E).

**Figure 8.**
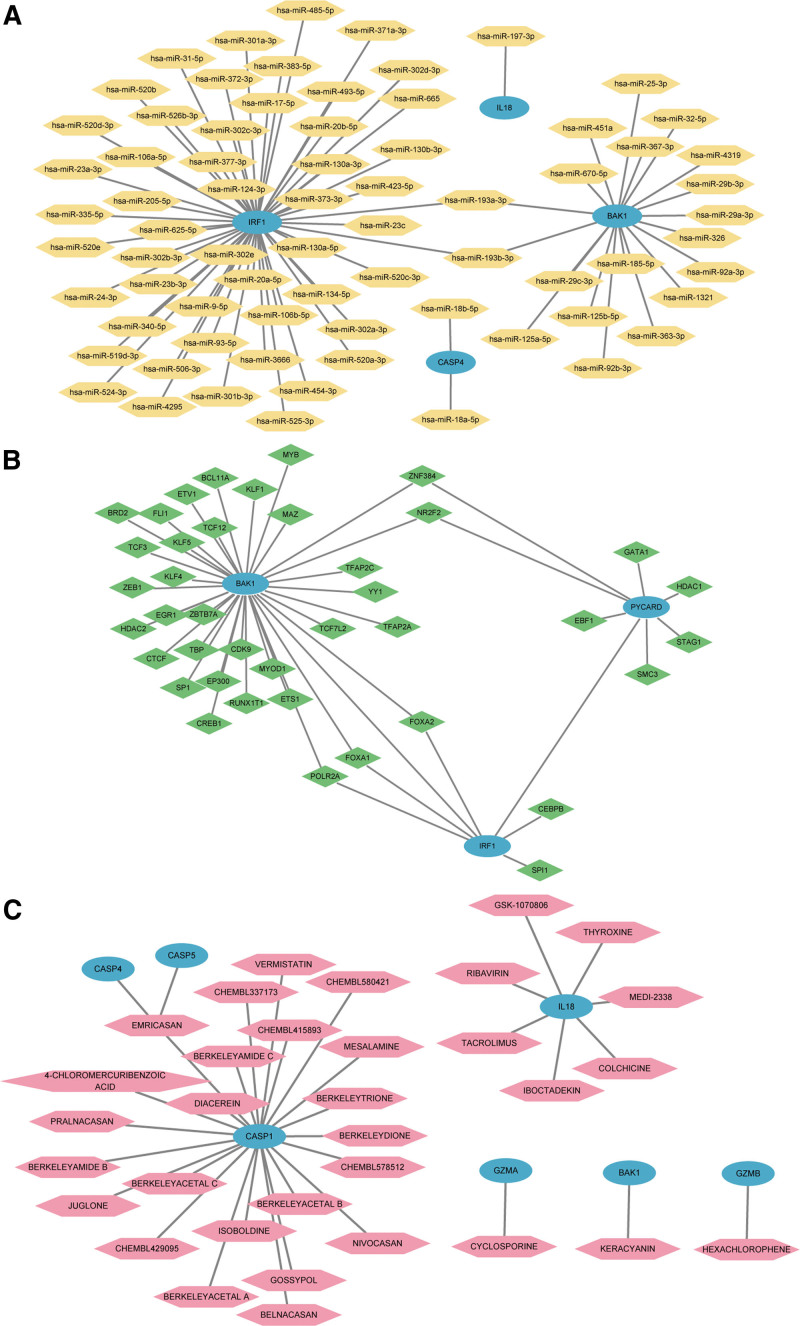
Interaction network of mRNA-miRNA, mRNA-TF, mRNA-drugs of hub genes in PPI network of PANoptosis-related differentially expressed gene (PDGs). Interaction network of mRNA-miRNA (A), mRNA-TF (B), mRNA-drugs (C) of hub genes in PPI network of PDGs. The sky blue oval block in mRNA-miRNA network (A) represents mRNA. The orange hexagonal block represents miRNA. The sky blue oval block in mRNA-TF (B) network represents mRNA. Green diamond represents TF. The sky blue oval block represents in mRNA-drugs (C) mRNA. The pink hexagonal block represents drug. PPI network = protein-protein interaction network; TF = transcription factors.

We applied mRNA-miRNA data in the miRDB database to obtain potential miRNAs interacting with the 10 PDGs, and drawn mRNA-miRNA regulatory network by Cytoscape for visualization (Fig. 8A). The sky blue oval block in the network represents mRNA, and the yellow hexagonal blocks means miRNA. The network is composed of 4 hub genes (BAK1, CASP4, IL18, IRF1) and 72 miRNA, which contributed to 74 mRNA-miRNA interaction pairs. The CHIPBase database and the hTFtarget database were used to predict TF binding to the10 hub genes. We downloaded relevant data from the 2 databases and took the intersection between them and the 10 hub genes. Finally, we got an interaction relationship network of 3 hub genes (BAK1, IRF1, PYCARD) and 41 TF. Network visualization was performed with Cytoscape (Fig. 8B). The sky blue oval block represented mRNA, and the green diamonds are TF. BAK1 showed a high interaction rate with other TF and contributed to 34 mRNA-TF interaction pairs.

The drug gene interaction database was used to identify potential drug compounds of 10 PDGs. Finally, we got 34 drug compounds that corresponded to 7 PDGs (BAK1, CASP1, CASP4, CASP5, GZMA, GZMB, IL18) (Fig. 8C). Notably, there are 25 drugs or molecular compounds targeting CASP1. As was presented, the sky blue oval block means mRNA and the pink hexagonal block represents drugs.

### 3.6. Clinical correlation analysis of PDGs

**Figure 9.**
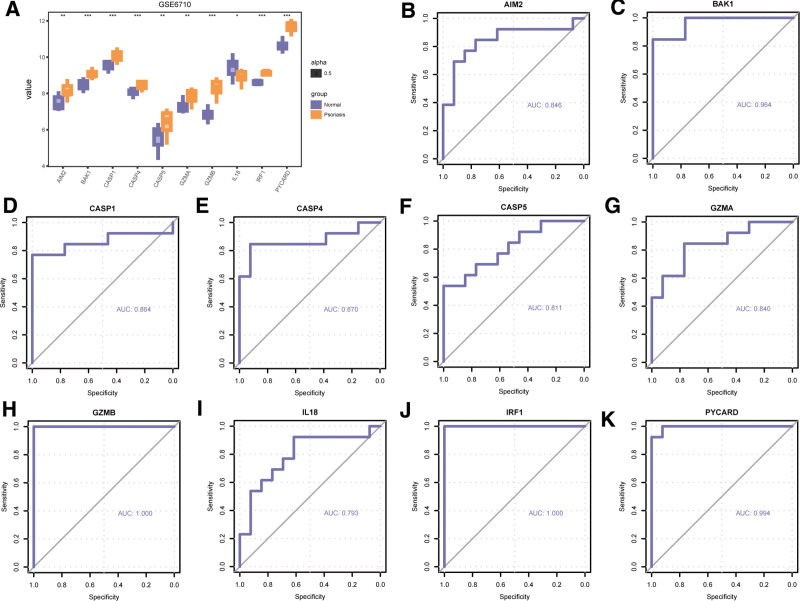
Differential expression analysis PDGs in GSE6710. (A) Expression difference of PDGs between psoriasis and normal group in GSE6710. (B) The ROC curve of AIM2 (B), BAK1 (C), CASP1 (D), CASP4I (E), CASP5 (F), GZMA (G), GZMB (H), IL18 (I), IRF1 (J), PYCARD (K) in GSE6710. ns *P *≥ .05, *P < .05, **P < .01, ***P < .001. The closer the area under the ROC curve (AUC) in the ROC curve is to 1, the better the diagnostic effect is. When AUC was 0.5 to 0.7, the accuracy was low. AUC between 0.7 and 0.9 had a certain accuracy; AUC > 0.9 had high accuracy. PDGs = PANoptosis-related differentially expressed genes; ROC = receiver operating characteristic.

To further explore the expression difference of the 10 PDGs, the correlation between the expression of these PDGs in the dataset and different groups was further analyzed. Firstly, we analyzed expression difference about the 10 PDGs in the psoriasis and normal group in GSE6710 using Wilcoxon signed rank test. We found significant expression difference of these genes existed between the psoriasis and normal group (Fig. 9A). (AIM2 *P *< .01; BAK1 *P *< .001, CASP1 *P *< .001, CASP4 *P *< .001, CASP5 *P *< .01, GZMA *P *< .01, GZMB *P *< .001, IL18 *P *< .05, IRF1 *P *< .001, PYCARD *P *< .001). Then, we plotted ROC curves for these genes, and the results were presented in Figure 9B–K. According to ROC curves, the expression of the 10 PDGs were significantly correlated with the occurrence of psoriasis. AUC for these PDGs were all above 0.7. Our findings were as follows: AIM2 (AUC = 0.846, Fig. 9B), BAK1 (AUC = 0.964, Fig. 9C), CASP1 (AUC = 0.864, Fig. 9D), CASP4 (AUC = 0.870, Fig. 9E), CASP5 (AUC = 0.811, Fig. 9F), GZMA (AUC = 0.840, Fig. 9G), GZMB (AUC = 1.000, Fig. 9H), IL18 (AUC = 0.793, Fig. 9I), IRF1 (AUC = 1.000, Fig. 9J), PYCARD (AUC = 0.944, Fig. 9K). To sum up, the 10 PDGs were significantly correlated with psoriasis occurrence.

**Figure 10.**
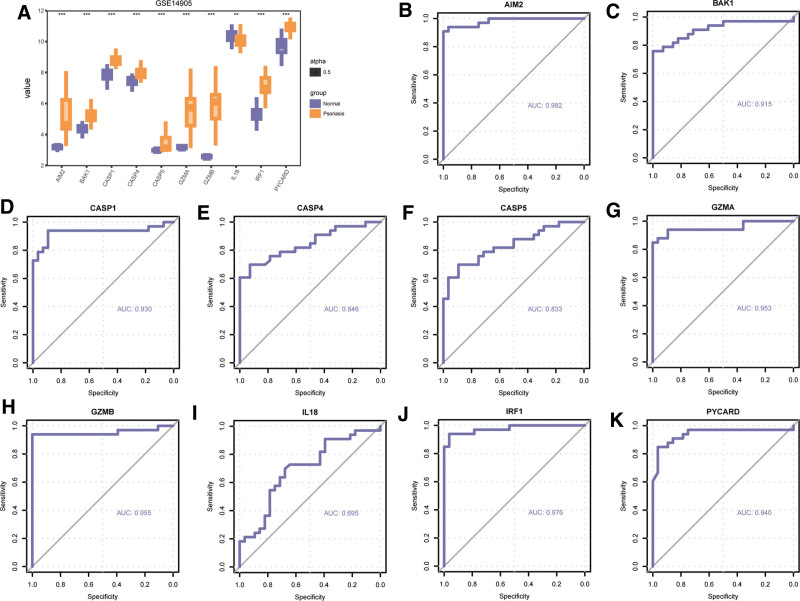
Differential expression analysis PDGs in GSE14905. (A) Expression difference of PDGs between psoriasis and normal group in GSE14905. (B) The ROC curve of AIM2 (B), BAK1 (C), CASP1 (D), CASP4 (E), CASP5 (F), GZMA (G), GZMB (H), IL18 (I), IRF1 (J), PYCARD (K) in GSE14905. ns *P *≥ .05, *P < .05, **P < .01,***P < .001. The closer the area under the ROC curve (AUC) in the ROC curve is to 1, the better the diagnostic effect is. When AUC was 0.5–0.7, the accuracy was low. AUC between 0.7 and 0.9 had a certain accuracy; AUC > 0.9 had high accuracy. PDGs = PANoptosis-related differentially expressed genes; ROC = receiver operating characteristic.

We conducted the same analysis on expression differences of the 10 PDGs in the GSE14905 dataset and GSE30999. In GSE14905, the expression of the 10 PDGs in the psoriasis and normal group had a significant difference (Fig. 10A). The results were as follows: AIM2 (*P *< .001), BAK1 (*P *< .001), CASP1 (*P *< .001), CASP4 (*P *< .001), CASP5 (*P *< .001), GZMA (*P *< .001), GZMB (*P *< .001), IL18 (*P *< .001), IRF1 (*P *< .001), PYCARD (*P *< .001). The ROC curves of 10 PDGs in the GSE14905 were also plotted and presented (Fig. 10B–K). According to the ROC curve, most gene expression were significantly correlated with the occurrence of psoriasis, except IL18. The results of AUC were as follows: AIM2 (AUC = 0.982, Fig. 10B), BAK1 (AUC = 0.915, Fig. 10C), CASP1 (AUC = 0.930, Fig. 10D), CASP4 (AUC = 0.846, Fig. 10E), CASP5 (AUC = 0.833, Fig. 10F), GZMA (AUC = 0.953, Fig. 10G), GZMB (AUC = 0.955, Fig. 10H), IL-18 (AUC = 0.695, Fig. 10I), IRF1 (AUC = 0.976, Fig. 10J), PYCARD (AUC = 0.940, Fig. 10K).

**Figure 11.**
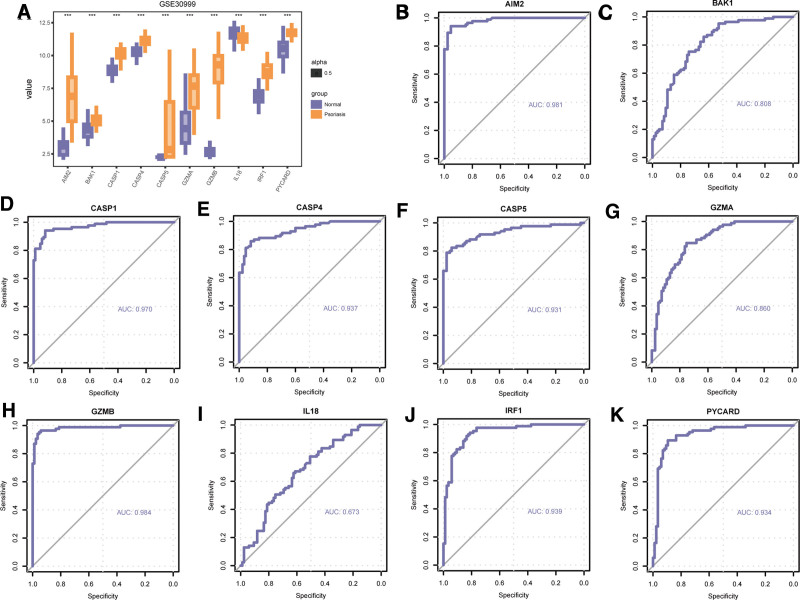
Differential expression analysis PDGs in GSE30999. (A) Expression difference of PDGs between psoriasis and normal group in GSE30999. (B) The ROC curve of AIM2 (B), BAK1 (C), CASP1 (D), CASP4 (E), CASP5 (F), GZMA (G), GZMB (H), IL18 (I), IRF1 (J), PYCARD (K) in GSE30999. ns *P *≥ .05, * *P *< .05, ** *P *< .01,*** *P *< .001. The closer the area under the ROC curve (AUC) in the ROC curve is to 1, the better the diagnostic effect is. When AUC was 0.5 to 0.7, the accuracy was low. AUC between 0.7 and 0.9 had a certain accuracy; AUC > 0.9 had high accuracy. PDGs = PANoptosis-related differentially expressed genes; ROC = receiver operating characteristic.

We explored the difference in expression of the 10 PDG between psoriasis and normal groups in GSE30999 based on Wilcoxon signed rank test. Results illustrated that there was significant expression difference of the 10 PDGs between the disease and normal groups in GSE30999 (Fig. 11A). The results were that AIM2 (*P *< .001), BAK1 (*P *< .001), CASP1 (*P *< .001), CASP4 (*P *< .001), CASP5 (*P *< .001), GZMA (*P *< .001), GZMB (*P *< .001), IL18 *P *< .001), IRF1 (*P *< .001), PYCARD (*P *< .001). The ROC curves of the 10 PDGs were presented in Figure 11B–K. According to ROC, most expression these PDGs were significantly related with the occurrence of psoriasis, except IL18. The AUC of these genes were as follows: AIM2 (AUC = 0.981, Fig. 11B), BAK1 (AUC = 0.808, Fig. 11C), CASP1 (AUC = 0.970, Fig. 11D), CASP4 (AUC = 0.937, Fig. 11E), CASP5 (AUC = 0.931, Fig. 11F), GZMA (AUC = 0.860, Fig. 11G), GZMB (AUC = 0.984, Fig. 11H), IL18 (AUC = 0.673, Fig. 11I), IRF1 (AUC = 0.939, Fig. 11J), PYCARD (AUC = 0.934, Fig. 11K).

### 3.7. Immune infiltration analysis

**Figure 12.**
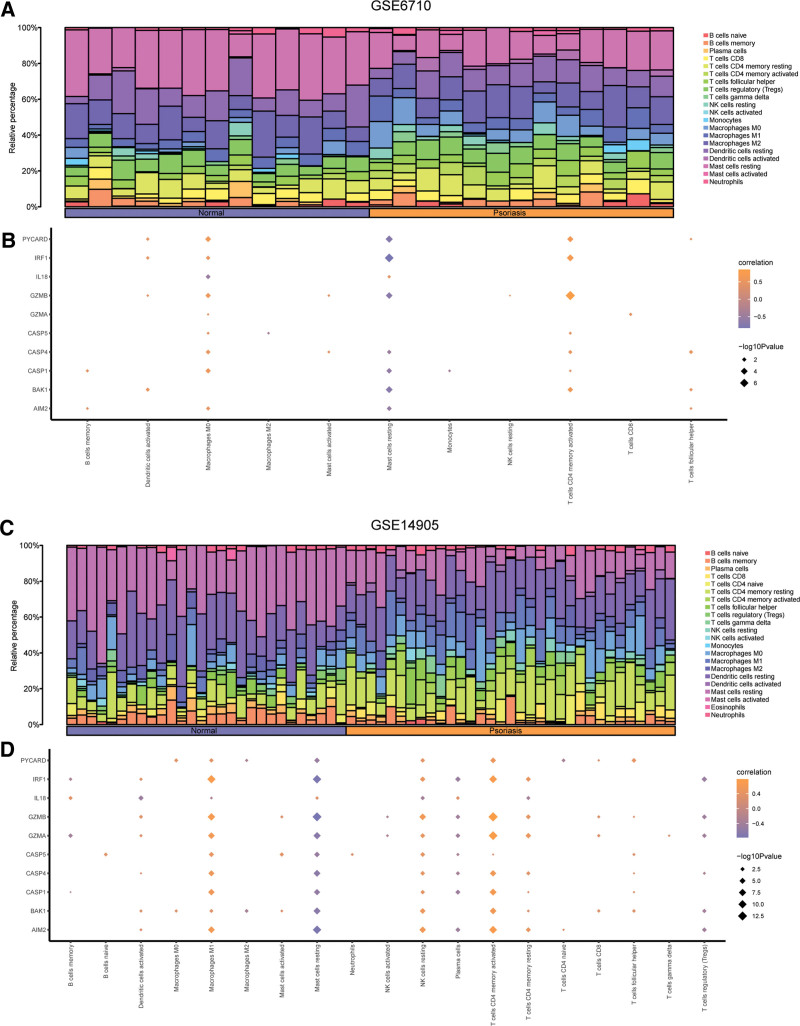
Immune infiltration analysis of GSE6710 and GSE14905. (A) The immune infiltration results of 22 immune cells in GSE6710. (B) Correlation between immune cells and PDGs in GSE6710. (C) The immune infiltration results of 22 immune cells in GSE14905. (D) Correlation between immune cells and PDGs in GSE14905. PDGs = PANoptosis-related differentially expressed genes.

Immune infiltration analysis was conducted in GSE6710, GSE14905 and GSE30999. We explored the interrelationship between 22 immune cells and gene expression profile of samples in GSE6710. The results were illustrated in bar graph (Fig. 12A). Furthermore, we explored the interrelationship between immune cell infiltration abundance and PDGs in samples of GSE6710. *P* < .05 was used as the screening criterion. We found significant correlation between the abundance of 11 immune cell infiltration and 10 PDGs expression in GSE6710 (*P *< .05), especially Macrophages M0 and CASP1, GZMB, IL18, PYCARD; Mast cells resting and BAK1, GZMB, IRF1, PYCARD; T cells CD4 memory activated and BAK1, GZMB, IRF1, PYCARD (Fig. 12B). Next, we executed the same research in the GSE14905 and GSE30999. The results of immune infiltration in samples of GSE14905 was presented in Figure 12C. The interrelationship between the abundance of immune cell infiltration and 0 PDGs expression were also calculated (Fig. 12D). The screen criterion was *P* < .05. We found significant interrelationship of 19 immune cell infiltration and expression of 10 PDGs in GSE14905 (*P *< .05), especially Macrophages M1 and AIM2, GZMB, IRF1; Mast cells resting and AIM2, GZMB, IRF1; NK cells resting and AIM2, GZMB; T cells CD4 memory activated and AIM2, GZMA, GZMB, IRF1 (Fig. 12D).

**Figure 13.**
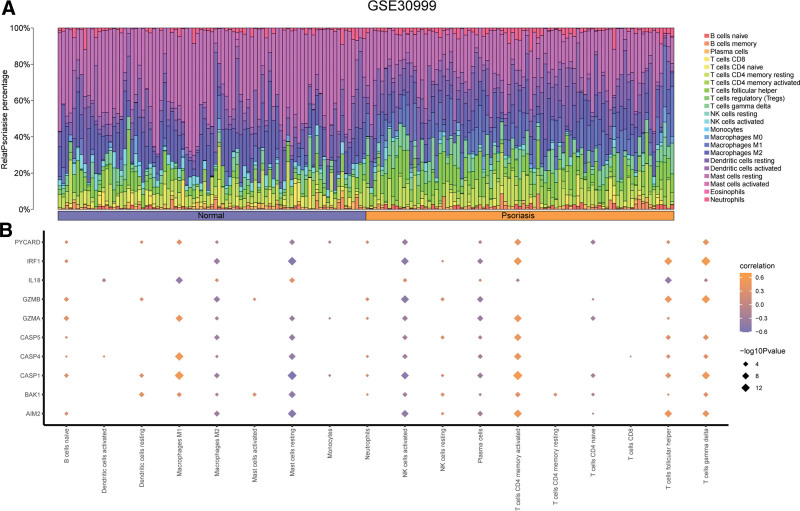
Immune infiltration analysis of GSE30999. (A) The immune infiltration results of 22 immune cells in GSE30999. (B) Correlation between immune cells and PDGs in GSE30999. PDGs: PANoptosis-related differentially expressed genes.

We used the CIBERSORT package and the Pearson algorithm to calculate the interrelationship between the expression profiles of all samples and the infiltration of 22 immune cells in GSE30999. The results were presented in Figure 13A. Additionally, we explored the relationship of the abundance of 22 immune cells and 10 PDGs in GSE30999. *P *<* *.05 was used as the screening criterion (Fig. 13B). We found significant correlation of the abundance of 18 immune cell infiltration and the 10 PDGs expression in normal groups (*P *< .05), especially Macrophages M1 and CASP1, CASP4; Mast cells resting and AIM2, CASP1, IRF1; NK cells activated and AIM2, CASP1, GZMB, IRF1; T cells CD4 memory activated and CASP1 (Fig. 13B).

## 4. Discussion

Psoriasis is a chronic, age-irrelevant, papulosquamous skin disease, especially in the ages of 18 to 39 years and 50 to 69 years.^[37]^ It imposes a heavy burden on individuals and society, and is related to multiple comorbidities (e.g., depression, psoriatic arthritis, and cardiometabolic syndrome).^[3]^ The pathogenesis of psoriasis is complex, including many factors like genetic, immunology and environment. Both adaptive and innate immune cells have essential role in pathogenesis of psoriasis, especially keratinocyte, dendritic cell, and T cell.^[38]^ Many abnormalities occur in psoriasis development, involving antigen presentation, activation of the NF-κB signaling pathway, differentiation of T helper cell populations, particularly Th17 cells, and increased IL-17 responses. The disease has no cure. Although biologicals could rapidly alleviate psoriasis, they still have no effect on its recurrence. Moreover, a variety of risk factors might trigger psoriasis. Despite substantial research, the etiology and pathogenesis of psoriasis are still unknown.

Recently, studies confirmed that PCD is closely related to the development of psoriasis.^[39]^ Researchers demonstrated that infliximab not only has anti-inflammatory effects in treating psoriasis, but also is associated with the induction of caspase-independent KC PCD in skin lesions.^[40]^ Proteomic analysis confirmed the activation of apoptotic pathways in psoriasis.^[39]^ In addition, ferroptosis and pyroptosis were also implicated in the pathogenesis of psoriasis.^[11]^ PANoptosis has molecules essential for pyroptosis, apoptosis, and necroptosis, which could activate different PCD pathways.^[41]^ As a highly interconnected PCD with mixture features of pyroptosis, apoptosis, and/or necroptosis, PANoptosis is also implicated in several inflammatory diseases like multiple sclerosis and rheumatoid arthritis.^[42]^ Nonetheless, the role of PANoptosis in psoriasis pathogenesis remains unclear.

PANoptosis is a newly defined PCD, and has become a new research hotspot. PANoptosis is regulated by sensors and signaling cascades to assemble multimolecular complexes to activate downstream molecules. Studies have identified ZBP1, AIM2 and RIPK1 were key molecules that promote PANoptosomes assembly and drive PANoptosis.^[43]^ Compared with these results, our study confirmed the close relationship between PANoptosis and the pathogenesis of psoriasis. Additionally, we identified 10 key genes including AIM2, BAK1 and CASP1, and confirmed the accuracy of these key genes in diagnosing psoriasis. We found pyroptosis and apoptosis signaling pathways were activated. Besides, we successfully constructed a network of PDGs in psoriasis, and confirmed that PANoptosis in psoriasis is tightly bound to the immune microenvironment. As far as we know, this is the first bioinformatics research revealing the presence of PANoptosis in the etiology of psoriasis.

In this study, we obtained 10 hub genes, namely, AIM2, BAK1, CASP1, CASP4, CASP5, GZMA, GZMB, IL18, IRF1, PYCARD. The AUC values of most genes were >0.8 in the 3 datasets apart from IL-18, demonstrating they were accurate and specific in differentiating psoriasis cases from healthy samples. Studies have identified and confirmed the role of pyroptosis-related DEGs in psoriasis like AIM2 and GZMB, AIM2, CASP4, and CASP5 using bioinformatics.^[44]^ In line with previous studies, the above genes were covered in our results, the reason maybe that PANoptosis has a mixture feature of PCD. Furthermore, our study provided more specific hub genes for PANoptosis in psoriasis. Absent in melanoma 2 (AIM2) is a cytosolic innate immune sensor and regulator that fosters the assembling of PANoptosomes and activate PANoptosis.^[43]^ Moreover, AIM2 is also critical for the development of psoriasis, where it exerts a pro-inflammatory effect by mediating cell death pathways like pyroptosis, which promotes the release of upstream pro-inflammatory cytokines (e.g., IL-1β and IL-18) and ultimately activates the IL-23/IL-17 axis.^[45]^ IL-17 has a significant role in the pathogenesis of psoriasis and was able to amplify IRF1 signals,^[46]^ meanwhile, IRF1 act as an upstream modulator of PANoptosis which could ultimately drive PANoptosis.^[47]^ CASP1 was a key molecule between PANoptosis and psoriasis. Upon stimulated with PANoptosome, CASP1 could induce the production of inflammatory cytokines of IL-1β and IL-18 that may directly facilitate the production of IL-17 and amplifies the inflammatory response of psoriasis.^[48]^ Granzyme B (GzmB) and granzyme A (GzmA) are 2 main subtypes of granzymes. The principal function of these granzymes is to induce target cell death like apoptosis or pyroptosis.^[49]^ Recent studies have shown that granzyme plays a pro-inflammatory role in psoriasis and that its expression is synergistically elevated with that of IL-23/IL-17-related genes.^[50]^ To sum up, the discovery of these genes adds to our understanding of the etiology and pathogenesis of psoriasis, as well as the role of PANoptosis for immune contexture of psoriasis.

The results of the GSEA are consistent with the pathogenesis of psoriasis, and indicate the role of innate immunity in psoriasis. The activated pathways were mostly proinflammatory pathways and immunoregulatory interactions pathways between immune cells, such as the activation of IL-23 and IFN-α/β signaling pathways. Besides, we found apoptosis pathways were also activated. Several atypical NF-kB pathways were enriched and activated in psoriasis, such as NF-kB activation through FADD RIP-1 Pathway mediated by caspase 8 and10, TNFR2 and Dectin1 mediated noncanonical NF-kB pathway. Caspase-8 is a key protein of the PANoptosome and has been associated with psoriasis development.^[51]^ TNF-TNFR2 signaling pathway might promote extrinsic apoptosis and necroptosis,^[52]^ and has been significantly associated with psoriasis. Dealing with these factors provides new ideas for the treatment psoriasis. After stimulation, various related pathways could be activated and increase the generation of proinflammatory molecules like interferon I (IFN-I) and proinflammatory cytokines like IL-1 and TNF. Molecules released during the process stimulate the immune cell response and activate the nonspecific response system and induce psoriasis.^[53]^ These results underscore the role of innate immunity and PANoptosis in psoriasis.

Furthermore, we analyzed the correlation of immune cells and PDGs in psoriasis based on 3 datasets. In line with previous studies,^[54,55]^ correlation analysis demonstrated PDGs was positively linked with proinflammatory infiltration of immune cells (e.g., Macrophages M0 and M1, T cells CD4 memory activated), and negatively linked with anti-inflammatory (e.g., Macrophages M2) or regulatory (e.g., T cells regulatory) immune cell infiltrations, which might due to the psoriasis pathogenesis of high immune infiltration and abnormal proliferation of KC. Increasing evidence comfirmed that macrophages are also crucial to psoriasis pathogenesis.^[56,57]^ There are 2 subtypes of macrophages after polarization, which are MI and M2.^[58]^ The former could secret inflammatory factors (e.g., IL-1, IL-17A, and IL-23), and mainly playing a pro-inflammatory role in psoriasis. The latter could secrete inflammatory factors like IL-10, transforming growth factor- β, C-C motif chemokine ligand 17, and may contribute to anti-inflammatory effects of psoriasis.^[57]^ Evidence demonstrated that inhibiting the activation of M1 and promoting M2 polarization could affect the development of psoriasis.^[57]^ In coherence with prior work, our findings demonstrated PDGs in psoriasis were correlated with the direction of macrophage polarization, these genes demonstrated positive correlation with M1, and negative correlation with M2, suggesting that regulating the M1 and M2 polarization direction of macrophages may affect the development of psoriasis through the PANoptosis pathway.

There were some drawbacks in our study as well. Firstly, although we performed comprehensive bioinformatics analysis, the results need to be interpreted cautiously, because we did not perform experimental or clinical trials to validate. In addition, immune infiltration analysis was performed using transcriptome data. Thus, we were unable to confirm whether PANoptosis could lead to infiltration of immune cells, or immune cells participate in PANoptosis pathway. Thus, further studies are necessary to establish the precise mechanisms.

In summary, our study identified novel PDGs and activated pathways in psoriasis. We confirmed the accuracy of these genes in diagnosing psoriasis, and found the infiltration of proinflammatory immune cells was positively linked to PDGs expression, whereas infiltration of anti-inflammatory or regulatory immune cells was negatively linked. These results pave a new road for further studies on the etiology and pathogenesis of psoriasis, as well as the therapeutic options for psoriasis.

## Author contributions

**Conceptualization:** Lingling Lu, Aimin Liu.

**Data curation:** Buxin Zhang, Meiling Shi.

**Formal analysis:** Buxin Zhang, Meiling Shi.

**Methodology:** Lingling Lu.

**Supervision:** Lingling Lu, Aimin Liu.

**Validation:** Lingling Lu, Aimin Liu.

**Writing – original draft:** Lingling Lu.

**Writing – review & editing:** Meiling Shi, Aimin Liu.
